# Preoperative prognostic nutritional index as a predictive factor for postoperative pneumonia in esophageal cancer patients undergoing esophagectomy

**DOI:** 10.3389/fnut.2025.1674518

**Published:** 2025-10-28

**Authors:** Chen Chen, Chenglin Li

**Affiliations:** Department of Thoracic Surgery, The Affiliated Huaian No.1 People’s Hospital of Nanjing Medical University, Huaian, Jiangsu, China

**Keywords:** esophageal cancer, prognostic nutritional index, risk factors, nomogram model, predictive factors

## Abstract

**Background:**

Postoperative pneumonia (POP) remains a serious complication following esophagectomy for esophageal cancer (EC) patients, contributing to increased morbidity, mortality, and healthcare costs. This study aimed to evaluate whether preoperative prognostic nutritional index (PNI) could be an independent predictor of POP in EC patients.

**Methods:**

This study included 200 EC patients who underwent esophagectomy between January 2021 to December 2022. Receiver operating characteristic (ROC) curve analysis was conducted to assess the predictive ability of preoperative PNI for POP. Univariate and multivariate logistic regression analyses were used to identify risk factors for POP among EC patients. A predictive nomogram model was conducted. The performance of the nomogram model was evaluated by the AUC curve, calibration curve and decision curve analysis (DCA).

**Results:**

Two hundred EC patients receiving esophagectomy were included finally, and 73 (36.5%) cases developed POP. ROC curve analysis showed that preoperative PNI predicted the occurrence of POP with an AUC value of 0.602 at a cut-off value of 49.6; the sensitivity, specificity, and Youden index was 64.38%, 63.78%, 0.2716, respectively. Univariate logistic regression analysis showed that male, aged ≥60 years old, TNM stage III, tumor location, hospital stay time >16 days, WBC counts >5.62 × 10^9^/L, neutrophil counts >3.52 × 10^9^/L, monocyte counts >0.40 × 10^9^/L, and preoperative PNI ≤ 49.6 were risk factors for POP. Multivariate logistic regression analysis indicated that tumor location, hospital stay time >16 days, WBC counts >5.62 × 10^9^/L, monocyte counts >0.40 × 10^9^/L, and preoperative PNI ≤ 49.6 were significant risk factors for POP among EC patients receiving esophagectomy. A nomogram model was established. The ROC curve incorporating PNI showed an excellent discrimination in detecting POP with an AUC value of 0.831 (95% CI: 0.772–0.890). The calibration curve suggested that the predicted results of this nomogram model exhibited a good concordance with the actual results. The DCA indicated that this nomogram model achieved net benefits for predicting POP.

**Conclusion:**

Preoperative PNI is a significant predictive factor for the occurrence of POP in EC patients. The nomogram model incorporating preoperative PNI shows good accuracy and clinical practicality in predicting the occurrence of POP among EC patients.

## 1 Introduction

Esophageal cancer (EC), the seventh most common cancer globally, remains a formidable clinical challenge, with over 600,000 new cases and 540,000 deaths annually (1, 2). Despite advances in treatment, the prognosis of EC remains poor, with low five-year survival rates, underscoring the need for better risk stratification and perioperative management (3, 4). Up to now, surgery remains the most effective treatment for EC patients. Various postoperative complications for EC patients included anastomotic leakage, pneumonia, recurrent laryngeal nerve injury, gastric emptying disorders, anastomotic stenosis, chylothorax, etc. Postoperative pneumonia (POP) was one of the most common postoperative complications of EC. The incidence of POP in patients receiving esophagectomy ranged from 14.60 to 39.26% (5). POP was reported to be associated with prolonged hospitalization, increased mortality, and elevated healthcare costs (6). Therefore, early prediction of POP is important for surgeons to make suitable clinical decisions. Identifying predictive markers for patients with POP is urgently necessary.

Esophagectomy could lead to weight loss, inadequate food intake, and nutrition issues (7). Preoperative nutritional status was associated with an increased risk of postoperative adverse events and a poor prognosis for EC patients (8). A pre-treatment nutritional assessment is recommended according to the nutrition guidelines (7, 9). The prognostic nutritional index (PNI), derived from serum albumin and lymphocyte counts, has gained recognition as a biomarker integrating nutritional and immune status of patients. PNI was regarded as a nutritional marker to evaluate the malnutrition status of patients with EC (10). A host of studies demonstrated that PNI was associated with the occurrence of postoperative complications and the survival of EC patients after esophagectomy (11–16). However, its role in predicting POP after esophagectomy, a common complication with significant clinical and economic consequences, remains unclear. Therefore, this study aimed to investigate the association between preoperative PNI and the risk of POP in patients with EC. By elucidating this relationship, we determined to inform targeted preoperative optimization and reduce the burden of this life-threatening complication.

## 2 Methods and materials

### 2.1 Study design and setting

This study was a retrospective observational analysis. Patients who underwent esophagectomy were included from The Affiliated Huaian No.1 People’s Hospital of Nanjing Medical University between January 2021 to December 2022. Inclusion Criteria were as follows: 1. EC patients aged 40–90 years old; 2. EC patients received radical esophagectomy; 3. TNM stage was from I to III. EC patients with the following conditions were excluded: 1. patients with incomplete clinical data; 2. patients refused to participate in this study; 3. patients received preoperative use of anti-infective drugs; 4. infections occurred in other organs after surgery; 5. EC patients not undergoing surgical treatment. Written informed consent was provided by every patient. This study was approved by the Ethics committee of Huaian No.1 People’s Hospital (KY-2025-097-01). This study conformed to the Declaration of Helsinki guidelines.

### 2.2 Definition of POP

POP was diagnosed within 30 days after operation in one or both lungs infection according to the following items: (1) chest computed tomography scans or X-rays showing relevant imaging presentation, such as infiltrative lesions; (2) respiratory symptoms including fever, cough, and difficulty breathing were shown; (3) signs of lung consolidation were presented, such as pulmonary moist rales (17–19). Surgeons documented pneumonia occurrence through clinical assessments and radiological investigations.

### 2.3 Data collection

Age, sex, drinking, smoking, body mass index (BMI), diabetes mellitus, tumor node metastasis (TNM) stage, hypertension, tumor location, and hospital stay time were collected. Preoperative laboratory results including monocyte, white blood cell (WBC), neutrophil, platelet, albumin, and lymphocyte were extracted from electronic medical records. The PNI calculation required the following formula: PNI = albumin (g/L) + 5 × lymphocyte count (10^9^/L).

### 2.4 Statistical analysis

The continuous variables were presented as the means ± standard deviations (SDs), or medians with interquartile ranges (IQRs), depending on the data distribution patterns. Categorical variables were displayed as frequencies and percentages. The independent t-test or Mann–Whitney U test was used to analyze continuous variables, and the chi-square test or Fisher’s exact test was used to evaluate categorical variables for group comparisons. Receiver operating characteristic (ROC) curve analysis was used to assess the predictive ability of PNI for POP. This study involved univariate and multivariate logistic regression analyses to detect independent pneumonia predictors. The ROC curve, decision curve analysis (DCA), and calibration plots were used to evaluate the nomogram model. Odds ratio (OR) and 95% confidence intervals (CIs) were calculated. A *p*-value lower than 0.05 was considered statistically significant. SPSS version 22.0, GraphPad Prism 8, and R software 4.1.3 were used.

## 3 Results

### 3.1 Patient characteristics

**Figure 1:**
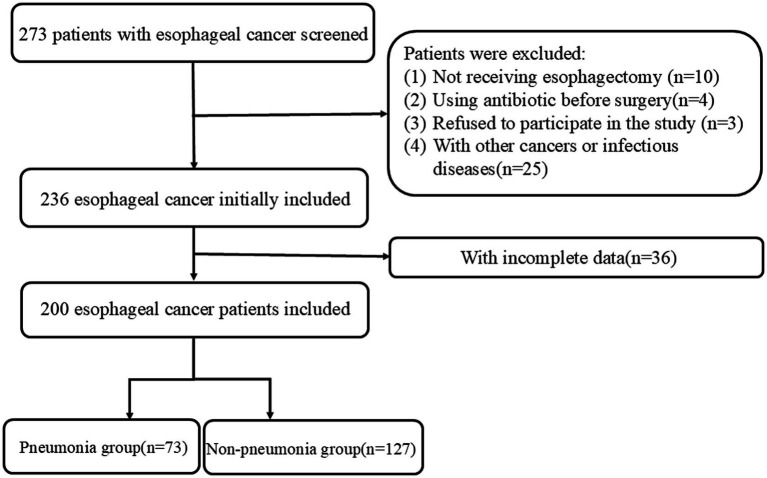
Flow diagram of enrolled patients.

A total of 200 patients with EC undergoing esophagectomy were included in this study (Figure 1). Seventy-three EC patients developed POP, and the incidence rate was 36.5%. Most of studies reported lower incidence of POP in EC patients (<36.5%) (19–23), but some showed higher incidence of POP (>36.5%) (24). Table 1 presents the clinicopathological characteristics of EC patients, comparing those in the non-pneumonia group (*n* = 127) with those in the pneumonia group (*n* = 73). Significant differences were observed in several variables. Male patients were more prevalent in the pneumonia group (78.1%) compared to the non-pneumonia group (63.8%) (*p* = 0.035). Patients aged 60 years or older were more common in the non-pneumonia group (89.8%) than in the pneumonia group (78.1%) (*p* = 0.024). TNM stage III was more frequently observed in the pneumonia group (47.9%) compared to the non-pneumonia group (32.3%) (*p* = 0.028). Tumor location also differed significantly between the pneumonia group and the non-pneumonia group (*p* = 0.015). Laboratory findings revealed that patients in the pneumonia group had significantly higher WBC counts (>5.62 × 10^9^/L, 65.8% vs. 40.2%, *p* < 0.001), neutrophil counts (>3.52 × 10^9^/L, 58.9% vs. 43.3%, *p* = 0.034), and monocyte counts (>0.40 × 10^9^/L, 65.8% vs. 34.6%, *p* < 0.001). Additionally, the pneumonia group had a lower PNI (PNI ≤ 49.6, 63.0% vs. 35.4%, *p* < 0.001). Hospital stay time was notably longer in the pneumonia group, with 56.2% of patients staying more than 16 days compared to 29.1% in the non-pneumonia group (*p* < 0.001). Other variables including BMI, smoking, drinking, hypertension, diabetes mellitus, platelet count, lymphocyte count, and albumin levels did not show statistically significant differences between the two groups.

**Table 1:** Clinicopathological characteristics of esophageal cancer patients between non-pneumonia and pneumonia groups.

Variables	Non-pneumonia group (*n* = 127)	Pneumonia group (*n* = 73)	*P*-value
Sex, n (%)			0.035*
Female	46 (36.2%)	16 (21.9%)	
Male	81 (63.8%)	57 (78.1%)	
Age, n (%)			0.024*
<60 years	13 (10.2%)	16 (21.9%)	
≥60 years	114 (89.8%)	57 (78.1%)	
BMI, n (%)			0.161
≤23.9 kg/m^2^	78 (61.4%)	52 (71.2%)	
>23.9 kg/m^2^	49 (38.6%)	21 (28.8%)	
Drinking, n (%)			0.732
No	115 (90.6%)	65 (89.0%)	
Yes	12 (9.4%)	8 (11.0%)	
Smoking, n (%)			0.851
No	104 (81.9%)	59 (80.8%)	
Yes	23 (18.1%)	14 (19.2%)	
Hypertension, n (%)			0.520
No	94 (74.0%)	57 (78.1%)	
Yes	33 (26.0%)	16 (21.9%)	
Diabetes mellitus, n (%)			0.770
No	118 (92.9%)	67 (91.8%)	
Yes	9 (7.1%)	6 (8.2%)	
TNM stage, n (%)			0.028*
I-II	86 (67.7%)	38 (52.1%)	
III	41 (32.3%)	35 (47.9%)	
Tumor location, n (%)			0.015*
Upper	12 (9.4%)	14 (19.2%)	
Middle	83 (65.4%)	33 (45.2%)	
Lower	32 (25.2%)	26 (35.6%)	
Hospital stay time, n (%)			<0.001*
≤16 day	90 (70.9%)	32 (43.8%)	
>16 day	37 (29.1%)	41 (56.2%)	
WBC, n (%)			<0.001*
≤5.62*10^9^/L	76 (59.8%)	25 (34.2%)	
>5.62*10^9^/L	51 (40.2%)	48 (65.8%)	
Neutrophil, n (%)			0.034*
≤3.52*10^9^/L	72 (56.7%)	30 (41.1%)	
>3.52*10^9^/L	55 (43.3%)	43 (58.9%)	
Monocyte, n (%)			<0.001*
≤0.40*10^9^/L	83 (65.4%)	25 (34.2%)	
>0.40*10^9^/L	44 (34.6%)	48 (65.8%)	
Platelet, n (%)			0.463
≤189.5*10^9^/L	61 (48.0%)	39 (53.4%)	
>189.5*10^9^/L	66 (52.0%)	34 (46.6%)	
Lymphocyte, n (%)			0.317
>1.46*10^9^/L	65 (51.2%)	31 (42.5%)	
≤1.46*10^9^/L	62 (48.8%)	42 (57.5%)	
Albumin, n (%)			0.145
≥42.8 g/L	71 (55.9%)	33 (45.2%)	
<42.8 g/L	56 (44.1%)	40 (54.8%)	
PNI, n (%)			<0.001*
>49.6	82 (64.6%)	27 (37.0%)	
≤49.6	45 (35.4%)	46 (63.0%)	

BMI, body mass index; TNM stage, tumor node metastasis stage; WBC, white blood cell; PNI, prognostic nutritional index. *indicating statistically significant (*P* < 0.05).

### 3.2 Predictive ability of PNI for POP

**Figure 2:**
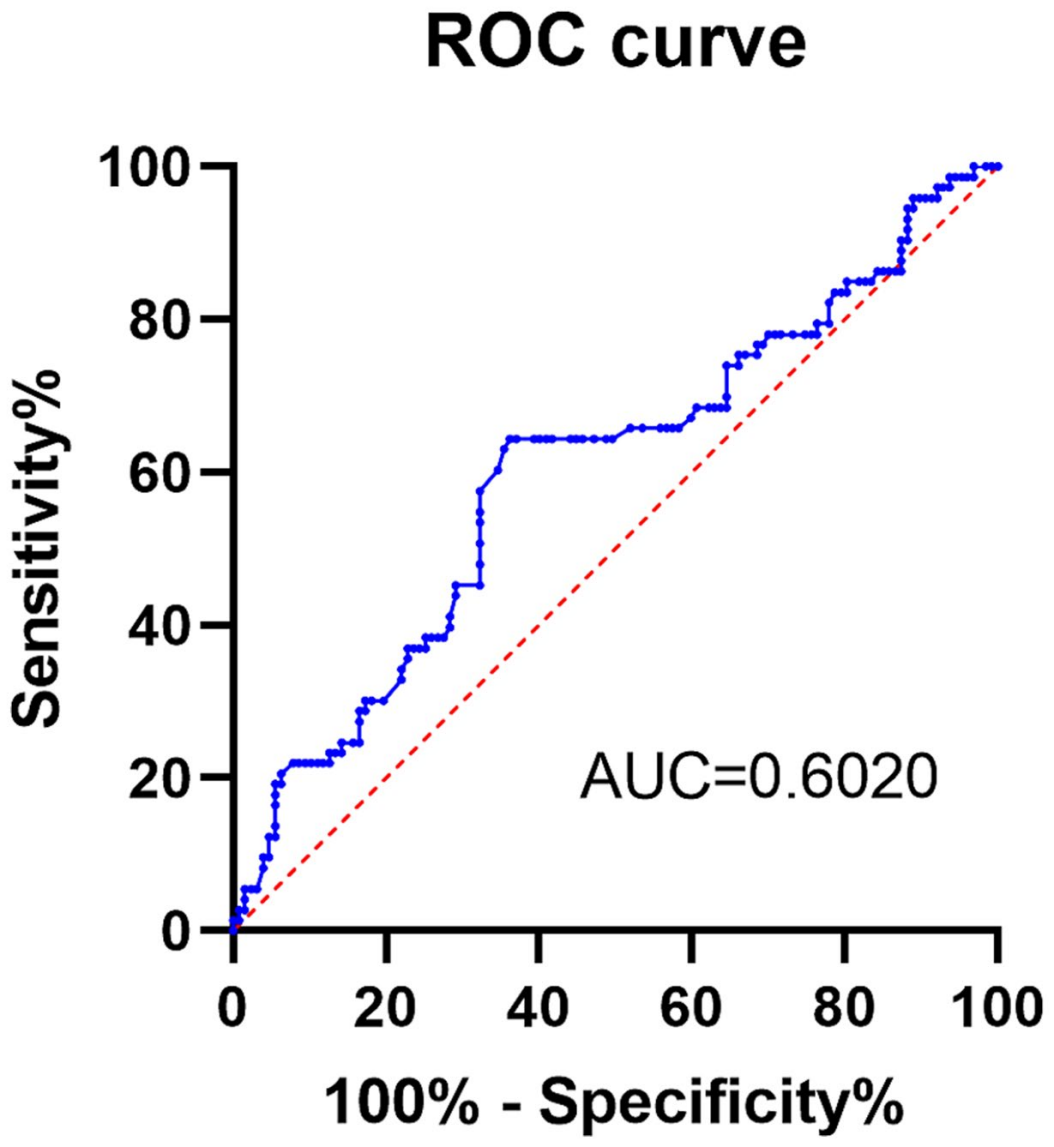
The receiver operating characteristic curve of preoperative PNI in predicting the occurrence of POP.

The predictive ability of preoperative PNI was evaluated in this study (Table 2). ROC curve analysis showed that PNI predicted the occurrence of POP with an AUC value of 0.602 at a cut-off value of 49.6. The sensitivity was 64.38%; the specificity was 63.78%; and the Youden index was 0.2716. In conclusion, the predictive ability of preoperative PNI for POP was moderate (Figure 2).

**Table 2:** Optimal cut-off value of PNI for predicting the postoperative pneumonia.

Variables	Cut-off value	Sensitivity %	Specificity %	AUC value	Youden index
PNI	*≤*49.6	64.38	63.78	0.6020 (0.5185–0.6855)	0.2716

PNI, prognostic nutritional index.

### 3.3 Risk factors of POP among EC patients

The univariate logistic regression analysis for identifying risk factors of POP in EC patients is summarized in Table 3. Male was associated with a significantly higher risk of POP compared to female (OR, 2.023; 95% CI, 1.043–3.923; *p* = 0.037). Patients aged 60 years or older had a lower risk compared to those under 60 years (OR, 0.406; 95% CI, 0.183–0.902; *p* = 0.027). Advanced TNM stage (stage III vs. I–II) was significantly associated with an increased risk of POP (OR, 1.932; 95% CI, 1.070–3.489; *p* = 0.029). Tumor location in the middle esophagus, compared to the upper esophagus, was associated with a reduced risk of POP (OR, 0.341; 95% CI, 0.143–0.814; *p* = 0.015). Prolonged hospital stay time (>16 days) was strongly associated with an increased risk of POP (OR, 3.117; 95% CI, 1.710–5.680; *p* < 0.001). Elevated WBC counts (>5.62 × 10^9^/L) were linked to a higher risk of POP (OR, 2.861; 95% CI, 1.571–5.212; *p* = 0.001), as were elevated neutrophil counts (>3.52 × 10^9^/L; OR, 1.876; 95% CI, 1.047–3.363; *p* = 0.035) and monocyte counts (>0.40 × 10^9^/L; OR, 3.622; 95% CI, 1.976–6.639; *p* < 0.001). A lower PNI (PNI ≤ 49.6) was associated with an increased risk of POP (OR, 3.105; 95% CI, 1.707–5.647; *p* < 0.001).

**Table 3:** Univariate logistic regression analyses for predicting postoperative pneumonia.

Variables	OR (95% CI)	*P*-value
Sex		
Male vs. female	2.023(1.043, 3.923)	0.037*
Age		
≥60 years vs. < 60 years	0.406(0.183, 0.902)	0.027*
BMI		
>23.9 kg/m^2^ vs. ≤23.9 kg/m^2^	0.643(0.346, 1.195)	0.163
Drinking		
Yes vs. no	1.179(0.458, 3.034)	0.732
Smoking		
Yes vs. No	1.073(0.513, 2.243)	0.851
Hypertension		
Yes vs. No	0.800(0.404, 1.581)	0.520
Diabetes mellitus		
Yes vs. No	1.174(0.400, 3.442)	0.770
TNM stage		
III vs. I-II	1.932(1.070, 3.489)	0.029*
Tumor location		
Middle vs. upper	0.341(0.143, 0.814)	0.015*
Lower vs. upper	0.696(0.275, 1.763)	0.445
Hospital stay time		
>16 day vs. ≤16 day	3.117(1.710, 5.680)	0.000*
WBC		
>5.62*10^9^/L vs. ≤5.62 *10^9^/L	2.861(1.571, 5.212)	0.001*
Neutrophil		
>3.52 *10^9^/L vs. ≤3.52*10^9^/L	1.876(1.047, 3.363)	0.035*
Monocyte		
>0.40*10^9^/L vs. ≤0.40*10^9^/L	3.622(1.976, 6.639)	0.000*
Platelet		
>189.5*10^9^/L vs. ≤189.5*10^9^/L	0.806(0.453, 1.434)	0.463
Lymphocyte		
≤1.46*10^9^/L vs. >1.46*10^9^/L	1.343(0.753, 2.396)	0.318
Albumin		
<42.8 g/L vs. ≥42.8 g/L	1.537(0.861, 2.742)	0.146
PNI		
≤49.6 vs. >49.6	3.105(1.707, 5.647)	0.000*

BMI, body mass index; TNM stage, tumor node metastasis stage; WBC, white blood cell; PNI, prognostic nutritional index. *Indicating statistically significant (*P* < 0.05).

Further multivariate logistic regression analysis was conducted (Table 4). EC in the middle esophagus was associated with a reduced risk of POP compared to the upper esophagus (OR, 0.182; 95% CI, 0.057–0.579; *p* = 0.004), and tumors in the lower esophagus also showing a reduced risk (OR, 0.251; 95% CI, 0.070–0.903; *p* = 0.034). Hospital stay time >16 days was strongly associated with an increased risk of POP (OR, 3.417; 95% CI, 1.647–7.089; *p* = 0.001). Elevated WBC count (>5.62 × 10^9^/L) was a significant risk factor for POP (OR, 3.827; 95% CI, 1.353–10.827; *p* = 0.011), as was elevated monocyte count (>0.40 × 10^9^/L; OR, 3.006; 95% CI, 1.384–6.529; *p* = 0.005). Preoperative PNI ≤ 49.6 was also a significant risk factor for POP, with an odds ratio of 4.659 (95% CI, 2.149–10.103; *p* < 0.001). Moreover, other variables, including sex, age, TNM stage, and neutrophil counts, were not significantly associated with the risk of POP. In total, tumor location, hospital stay time >16 days, WBC count >5.62 × 10^9^/L, monocyte count >0.40 × 10^9^/L, and low PNI ≤ 49.6 were significant risk factors for POP among EC patients receiving esophagectomy.

**Table 4:** Multivariate logistic regression analyses for predicting postoperative pneumonia.

Variables	OR (95% CI)	*P*-value
Sex		
Male vs. female	1.616(0.665, 3.929)	0.289
Age		
≥60 years vs. < 60 years	0.566(0.207, 1.549)	0.268
TNM stage		
III vs. I-II	1.160(0.557, 2.416)	0.692
Tumor location		
Middle vs. upper	0.182(0.057, 0.579)	0.004*
Lower vs. upper	0.251(0.070, 0.903)	0.034*
Hospital stay time		
>16 day vs. ≤16 day	3.417(1.647, 7.089)	0.001*
WBC		
>5.62*10^9^/L vs. ≤5.62 *10^9^/L	3.827(1.353, 10.827)	0.011*
Neutrophil		
>3.52 *10^9^/L vs. ≤3.52*10^9^/L	0.570(0.212, 1.535)	0.266
Monocyte		
>0.40*10^9^/L vs. ≤0.40*10^9^/L	3.006(1.384, 6.529)	0.005*
PNI		
≤49.6 vs. >49.6	4.659(2.149, 10.103)	0.000*

TNM stage, tumor node metastasis stage; WBC, white blood cell; PNI, prognostic nutritional index. *Indicating statistically significant (*P* < 0.05).

### 3.4 Construction a nomogram model for predicting POP

**Figure 3:**
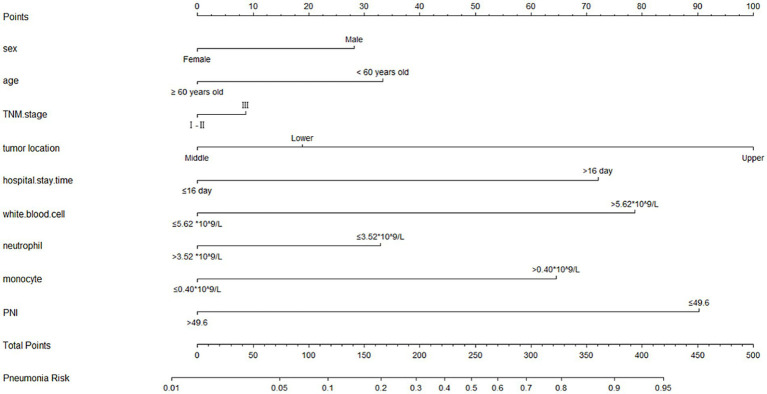
The nomogram for predicting occurrence of POP.

**Figure 4:**
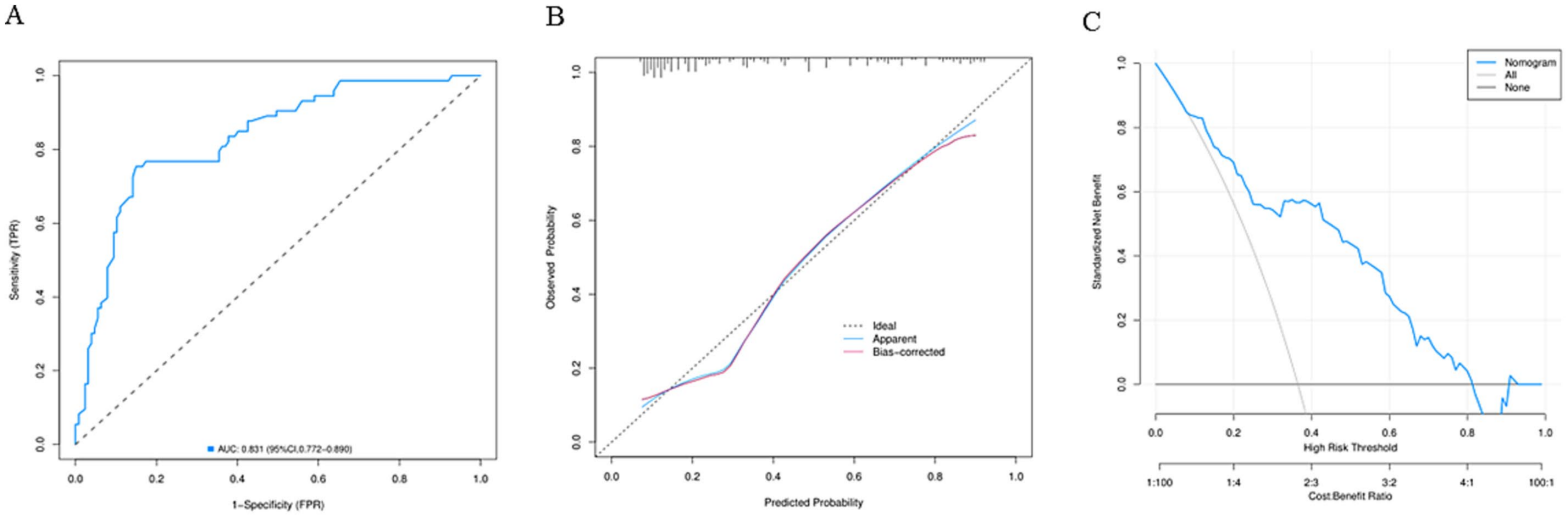
**(A)** The receiver operating characteristic curve of the nomogram model. **(B)** The calibration curve of the nomogram model. **(C)** The decision curve analysis of the nomogram model.

A nomogram model was established according the results of multivariate logistic regression analysis (Figure 3). The AUC value for this prediction model was 0.831 (95% Cl: 0.772–0.890), indicating that this nomogram model showed a good predictive validity for predicting POP (Figure 4A). The calibration curve of this nomogram model indicated that the nomogram predictive model presented a good consistency between the observational probability and predicted probability in predicting POP (Figure 4B). The DCA indicated that this nomogram model achieved the high clinical net benefit in predicting POP among EC patients (Figure 4C).

## 4 Discussion

### 4.1 Interpretation of main findings and comparison with previous studies

Our study demonstrated that preoperative PNI was a significant predictor of POP in patients with EC. This finding aligned with previous studies validating PNI as a reliable predictor of postoperative complications of EC patients. The discriminative ability of PNI confirmed that the utility of PNI in the risk stratification of POP, consistent with studies showing similar predictive performance for postoperative complications (12–14). Up to date, only one study explored the relationship between preoperative PNI and the risk of POP among EC patients receiving esophagectomy, and it showed that preoperative PNI was not a risk factor for POP (25), which was contradictory with this study. Some factors may contribute to this phenomenon. One, this study included EC patients with pneumonia during hospitalization after surgery, while the study by Nishimura et al. enrolled EC patients who occurred over 3 months after esophagectomy (25). Two, the sample sizes differed in these two studies. Three, different surgical methods and postoperative care may be potential reasons. In addition, some studies depicted the association between PNI and the clinical outcomes of pneumonia. A Japanese study indicated that the PNI could predict the risk of POP after lung cancer surgery (26), which was replicated in another study (27). Another Japanese study also showed that PNI was a risk factor for POP after general and digestive surgery (28). A Chinese study found that PNI was associated with mortality of COVID-19 patients (29). A study by Shang et al. showed that PNI was a significant predictor of new-onset pneumonia in peritoneal dialysis patients (30). Three studies indicated that PNI was negatively associated with the mortality of community-acquired pneumonia (31–33). According to these abovementioned studies, we could find a close relationship between PNI and the occurrence of pneumonia.

### 4.2 Possible mechanisms linking low PNI and the occurrence of POP

The association between low PNI and the occurrence of POP could be attributed to multiple interconnected mechanisms. Malnutrition, reflected by low PNI, impairs protein synthesis and tissue repair while weakening antioxidant defenses (34). Concurrently, lymphopenia compromises cellular immunity function, reducing the ability to combat respiratory pathogens (35). These effects are exacerbated by the systemic inflammatory response to surgery, which further suppresses immune function. The combination of nutritional deficiency and immune dysfunction created a vulnerable state where patients were more susceptible to pulmonary infections (36, 37). This mechanistic understanding was supported by studies showing that patients with low PNI have higher levels of inflammatory markers like CRP and PCT, which correlated with increased infection risk (38, 39). The specific mechanisms linking low PNI and the occurrence of POP requires further studies to explore.

### 4.3 Risk factors for POP other than PNI

Our analysis confirmed several established risk factors for POP. We found that tumor location, hospital stay time, WBC, and monocyte were also associated with the risk of POP. High level of WBC and monocyte before operation meant high inflammatory state in the body. In addition, the further aggravation of pulmonary inflammation caused by surgery made patients more prone to pulmonary infections. EC patients with longer hospital stay time had a greater likelihood of developing POP after surgery.

### 4.4 Clinical implications and recommendations

Malnutrition is substantially associated with higher morbidity, disability, delayed recovery, and elevated healthcare costs (40). Continuous monitoring of nutritional status of EC cancer patients is necessary, such as PNI. Routine preoperative PNI assessment should be implemented to identify high-risk patients who may benefit from nutritional optimization and immune support. According to the findings of this study, for patients with PNI ≤ 49.6, targeted interventions such as individualized nutritional therapy and prehabilitation programs may potentially reduce the risk of POP, which needs further studies to validate it. PNI could help clinical clinicians to evaluate risk stratification of POP. Preoperative PNI could help to identify patients with poor nutritional status. A meta-analysis indicated that the mixed nutrition therapy for postoperative esophageal cancer patients could reduce the incidence of postoperative complications including POP (41). Unfortunately, this study did not investigate whether preoperative nutritional intervention could reduce postoperative complications, including POP. We need to develop nutritional interventions to mitigate the adverse events of cancer-related malnutrition. It is pivotal to utilize markers to identify the POP in high-risk patients at an individual level when implementing nutritional interventions. The main challenge is to identify the most effective way to incorporate these markers into established assessment tools, optimizing personalized nutritional therapies for patients with malnutrition. Surprisingly, studies reported that artificial intelligence was a promising tool in healthcare with potential applications in nutritional management, which could help to improve early detection, risk stratification, and personalized nutritional therapies for EC patients with POP (42, 43).

### 4.5 Limitations of the study

Several limitations should be acknowledged. The retrospective design introduced potential selection bias and limited causal interpretation. Being a single-center study, our findings may have limited generalizability to other practice settings. The sample size, particularly in the pneumonia group, restricted our ability to analyze less common risk factors. Additionally, we could not analyze all potential confounders, such as variations in surgical technique or preoperative pulmonary function. Our study did not investigate whether preoperative nutritional interventions could reduce the incidence of POP in EC patients. These limitations highlighted the need for multicenter prospective studies to validate our findings.

## 5 Conclusion

In conclusion, our study finds that preoperative PNI as a valuable predictor of POP in EC patients. This strong association between low PNI and the risk of POP, along with the nomogram model’s excellent discriminatory ability, supports incorporating PNI into routine preoperative assessment. These findings underscore the importance of evaluating nutritional status and immune function before surgery and suggest that targeted preoperative optimization may reduce the risk of POP. Future research should focus on validating these results in larger, prospective cohorts and evaluating interventions to improve PNI in high-risk patients.

## Data Availability

The original contributions presented in the study are included in the article/supplementary material, further inquiries can be directed to the corresponding author.
